# Comparison of short-term efficacy and safety between total robotic and total 3D laparoscopic distal radical gastrectomy for gastric cancer in Enhanced Recovery After Surgery (ERAS) protocol: a propensity score matching study

**DOI:** 10.1007/s11701-023-01528-8

**Published:** 2023-01-11

**Authors:** Yuan Tian, Yecheng Lin, Chenyu Sun, Scott Lowe, Rachel Bentley, Peigang Yang, Honghai Guo, Pingan Ding, Zhidong Zhang, Dong Wang, Xuefeng Zhao, Yong Li, Qun Zhao

**Affiliations:** 1 Third Surgery Department, The Fourth Hospital of Hebei Medical University, No.12, Jian-Kang Road, Shijiazhuang, 050019 Hebei China; 2 AMITA Health Saint Joseph Hospital Chicago, 2900 N. Lake Shore Drive, Chicago, IL 60657 USA; 3 College of Osteopathic Medicine, Kansas City University, 1750 Independence Ave, Kansas City, MO 64106 USA

**Keywords:** Enhanced Recovery After Surgery, Robot, 3D-laparoscope, Distal gastrectomy

## Abstract

**Background:**

The application of Enhanced Recovery After Surgery (ERAS) protocol in gastrointestinal surgery has been widely accepted. The aim of this study was to compare the effect of ERAS in total robotic distal gastrectomy (TRDG) versus 3D total laparoscopic distal gastrectomy (3D-TLDG) for gastric cancer.

**Methods:**

We retrospectively evaluated 73 patients underwent TRDG and 163 patients who received 3D-TLDG. The propensity score was used for matching analysis according to a 1:1 ratio, so that there was no significant difference in the baseline data between the two groups. The short-term effect and safety of the two groups were compared.

**Results:**

The TRDG group had a less intraoperative bleeding (30.21 ± 13.78 vs. 41.44 ± 17.41 ml, *P* < 0.001), longer intraoperative preparation time (31.05 ± 4.93 vs. 15.48 ± 2.43 min, *P* < 0.001), shorter digestive tract reconstruction time (32.67 ± 4.41 vs. 39.78 ± 4.95 min, *P* < 0.001), shorter postoperative ambulation time (14.07 ± 8.97 vs. 17.49 ± 5.98 h, *P* = 0.007), shorter postoperative anal exhaust time (1.78 ± 0.79 vs. 2.18 ± 0.79 days, *P* = 0.003), shorter postoperative hospital stay (7.74 ± 3.15 vs. 9.97 ± 3.23 days, *P* < 0.001), lower postoperative pain score (*P* = 0.006) and higher hospitalization cost (89,907.15 ± 17,147.19 vs. 125,615.82 ± 11,900.80 RMB, *P* < 0.001) than the 3D-TLDG group.

**Conclusion:**

TRDG and 3D-TLDG under ERAS protocol are safe and feasible. Compared with 3D-TLDG, the TRDG has better intraoperative bleeding control effect and greater advantages in digestive tract reconstruction. After the combination of ERAS protocol, TRDG also has certain advantages in the recovery process of patients after surgery.

## Introduction

Although the incidence of esophageal-gastric junction cancer has been increasing in the past decades, the most common occurrence site of gastric cancer is still the antrum. Surgical resection remains the primary treatment for gastric cancer. Radical distal gastrectomy plays an important role in the prognosis of gastric antrum carcinoma [1, 2].

In recent years, minimally invasive surgery has developed rapidly. At present, 2D, 3D, and 4K laparoscopy and Da Vinci robot operating system have been applied to the surgical treatment of gastric cancer [3]. Previous studies have shown that laparoscopy as a safe and feasible procedure with better short-term efficacy than traditional open surgery in the treatment of gastric cancer [4]. Since *Hashizume *et al*.* [5] first reported robotic surgery for gastric cancer in 2002, various studies conducted around the globe have also shown the safety and advantages of robotic radical gastrectomy for gastric cancer [6–8]. As an optimized clinical management protocol, the Enhanced Recovery After Surgery (ERAS) is continuously applied to perioperative management of gastric cancer patients through multidisciplinary collaboration [9], which includes comprehensive preoperative education, avoidance of bowel preparation, effective analgesia, no drainage or nasogastric tube, early oral feeding, and immediate activity [10]. In addition, by reducing perioperative stress and complications with this approach, patients would have a better perioperative experience. At present, there is still a lack of research to compare the robotic surgery versus 3D laparoscopy in gastrointestinal surgery, and the safety of the two surgical approaches in ERAS protocol remains to be studied. Therefore, this study was conducted to explore the short-term efficacy and safety of total robotic distal gastrectomy (TRDG) versus 3D total laparoscopic distal gastrectomy (3D-TLDG) under ERAS protocol.

## Materials and methods

### Patients

A retrospective analysis of 298 patients with gastric cancer admitted to the Third Department of Surgery, Fourth Hospital of Hebei Medical University from January 2019 to December 2020 was performed. All patients underwent TRDG or 3D-TLDG under ERAS protocol. The clinicopathological data of the patients were analyzed and 62 patients who did not meet the criteria were excluded. The inclusion criteria were as follows: (1) The diagnosis of gastric cancer was confirmed by postoperative pathology, and the pathological stage was I–III according to UICC/AJCC guidelines; (2) Patients received TRDG or 3D-TLDG; (3) The digestive tract reconstruction methods were all Billroth II + Braun anastomosis; (4) The operations were performed by the same group of surgeons. The exclusion criteria were as follows: (1) Distant metastases to the liver, lung, peritoneum, etc., were found during the preoperative examination or intraoperative exploration; (2) Palliative surgical resection were conducted; (3) Neoadjuvant therapy (chemotherapy, targeted therapy, etc.) were given before surgery; (4) Tumors in other parts of the body were identified complications with tumors in other (5) Emergency surgery for bleeding, perforation, obstruction, etc. were conducted during the hospitalization.

**Fig. 1:**
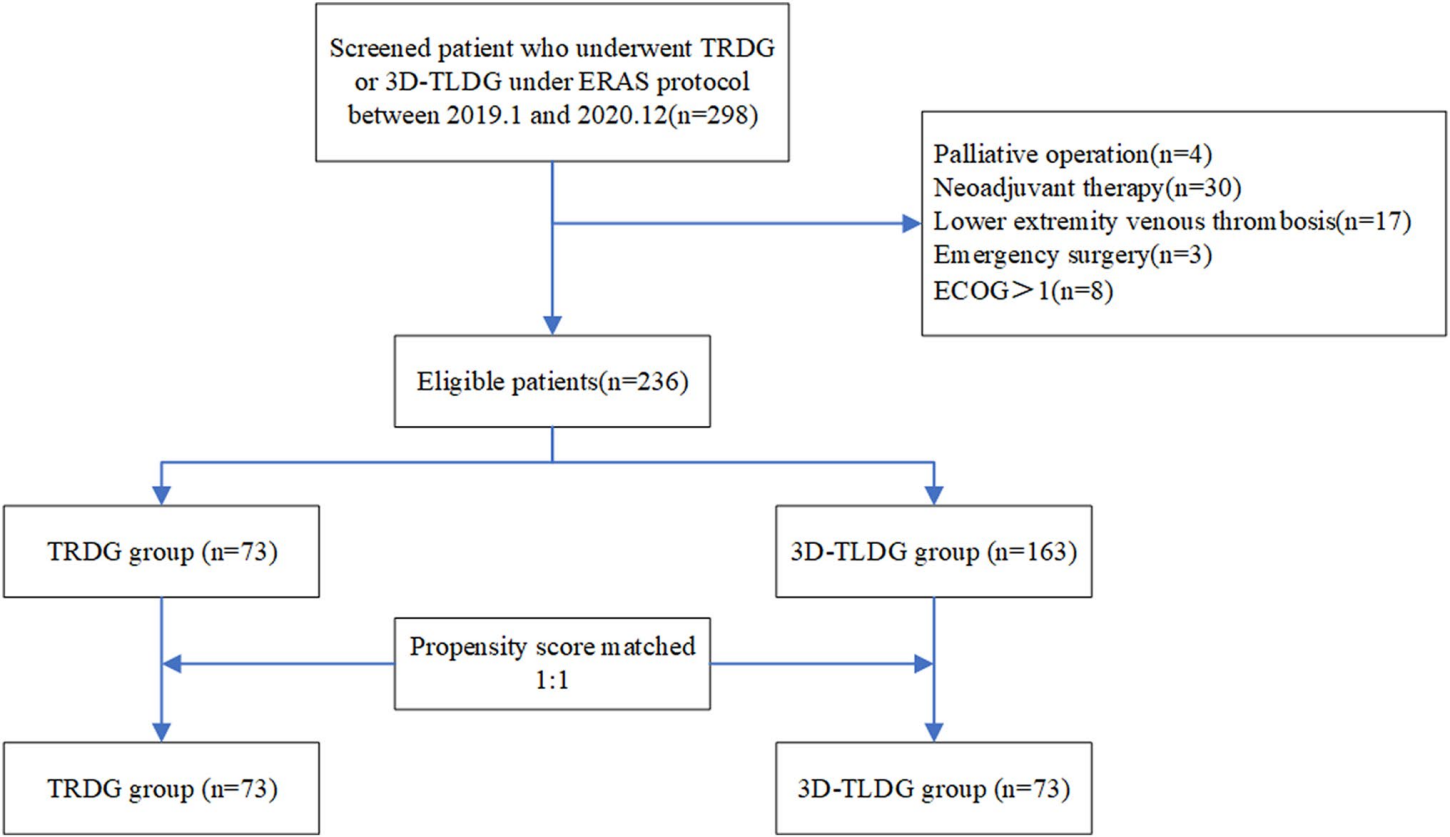
Patient enrollment process in this study

A total of 236 patients were finally included for retrospective study. Among them, 73 patients received TRDG under ERAS protocol, which was defined as the TRDG group, while the other 163 patients who received 3D-TLDG under ERAS protocol were considered as the 3D-TLDG group. The baseline data of the two groups were examined statistically, including gender, age, BMI, ECOG score, tumor size, Lauren classification, differentiation, and clinical and pathological stages. The propensity score was then used to match the baseline data of the two groups according to the ratio of 1:1. Finally, 73 cases in the TRDG group and 73 cases in the 3D-TLDG group were matched (Fig. 1). The baseline data of the two groups after matching in Table 1.

**Table 1:** The baseline characteristics in patients of two groups

	Entire cohort				Matched cohort			
	TRDG (*n* = 73)	3D-TLDG (*n* = 163)	*T*/*χ*^2^	*P*	TRDG (*n* = 73)	3D-TLDG (*n* = 73)	*T*/*χ*^2^	*P*
Gender								
Male	49	75	9.012	0.003	49	46	0.271	0.203
Female	24	88			24	27		
Age, year ± SD	55.92 ± 10.00	57.08 ± 10.76	− 0.784	0.434	55.92 ± 10.00	54.85 ± 11.47	0.600	0.549
BMI (kg/m^2^)	23.80 ± 2.87	23.61 ± 2.89	0.477	0.634	23.80 ± 2.87	23.87 ± 2.99	− 0.146	0.884
ECOG score								
0	39	109	3.898	0.048	39	34	0.685	0.408
1	34	54			34	39		
Size, cm ± SD	2.95 ± 1.36	3.08 ± 3.42	− 0.301	0.764	2.95 ± 1.36	2.82 ± 1.15	0.618	0.538
Differentiation								
Poor differentiation	32	62	0.740	0.691	32	31	0.136	0.934
Moderate differentiation	20	51			20	22		
Poor-to-moderate differentiation	21	50			21	20		
Lauren classification								
Intestinal	20	38	6.153	0.046	20	20	0.038	0.981
Diffuse	27	91			27	26		
Mixed	26	40			26	27		
Clinical stage								
I	32	73	0.441	0.802	32	37	0.917	0.632
II	24	58			24	23		
III	17	32			17	13		
Pathological stage								
I	36	75	2.484	0.289	36	39	0.247	0.884
II	13	44			13	12		
III	24	44			24	22		

## Methods

### Key points of ERAS protocol

1. Preoperative ERAS-related health education, including nutritional support, pain management and early ambulation;
2. No preoperative bowel preparation. Fasting for 5–6 h before operation, oral administration of less than 500 ml 10% glucose solution 2 h before operation. No indwelling gastrointestinal decompression tube before operation;
3. After general anesthesia, preoperative ultrasound-guided transversus abdominis plane (TAP) block analgesia was also conducted. At the same time, the patients were given heat preservation blanket and other related equipment to maintain the body temperature at 36–37 °C and room temperature at 25 °C;
4. Nasogastric tube was placed intraoperatively to reduce tissue edema, and abdominal drainage tube was also placed;
5. The postoperative pain was self-controlled with patient-controlled analgesia (PCA).
6. Encouraged patients to resume activity as tolerated and to ambulate early after operation;
7. Resumed clear liquid diet as early as possible and advanced as tolerated;
8. Nasogastric tube (NGT), catheters, and abdominal drainage tube were removed as early as possible.

### Surgery

**Fig. 2:**
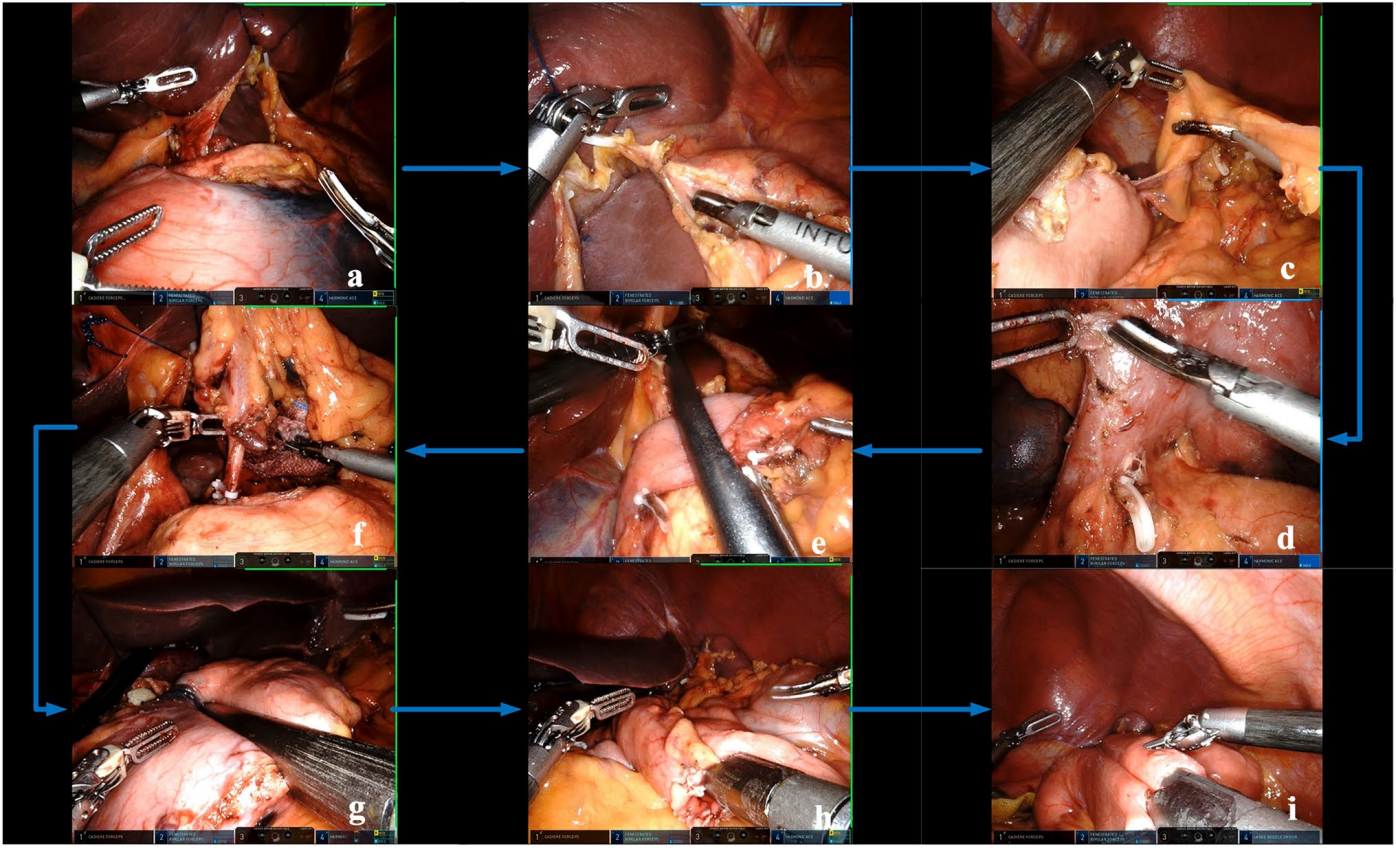
Robotic Surgical procedures. **a** The RGA&V were dissected, and No. 5 and 12a were dissected; **b** Dissociate the anterior wall of lesser curvature of stomach, and No. 1 and 3 were dissected; **c** The LEGA&V were dissected, and No. 4sb and 4d were dissected; **d** The RGEA&V dissected, and No.6 were dissected; **e** The duodenum was dissected; **f** No. 7, 8a, 9, and 11p were dissected; **g** Determine the upper margin to separate the gastric wall; **h** Billroth II anastomosis; **i** Braun’s anastomosis

**Fig. 3:**
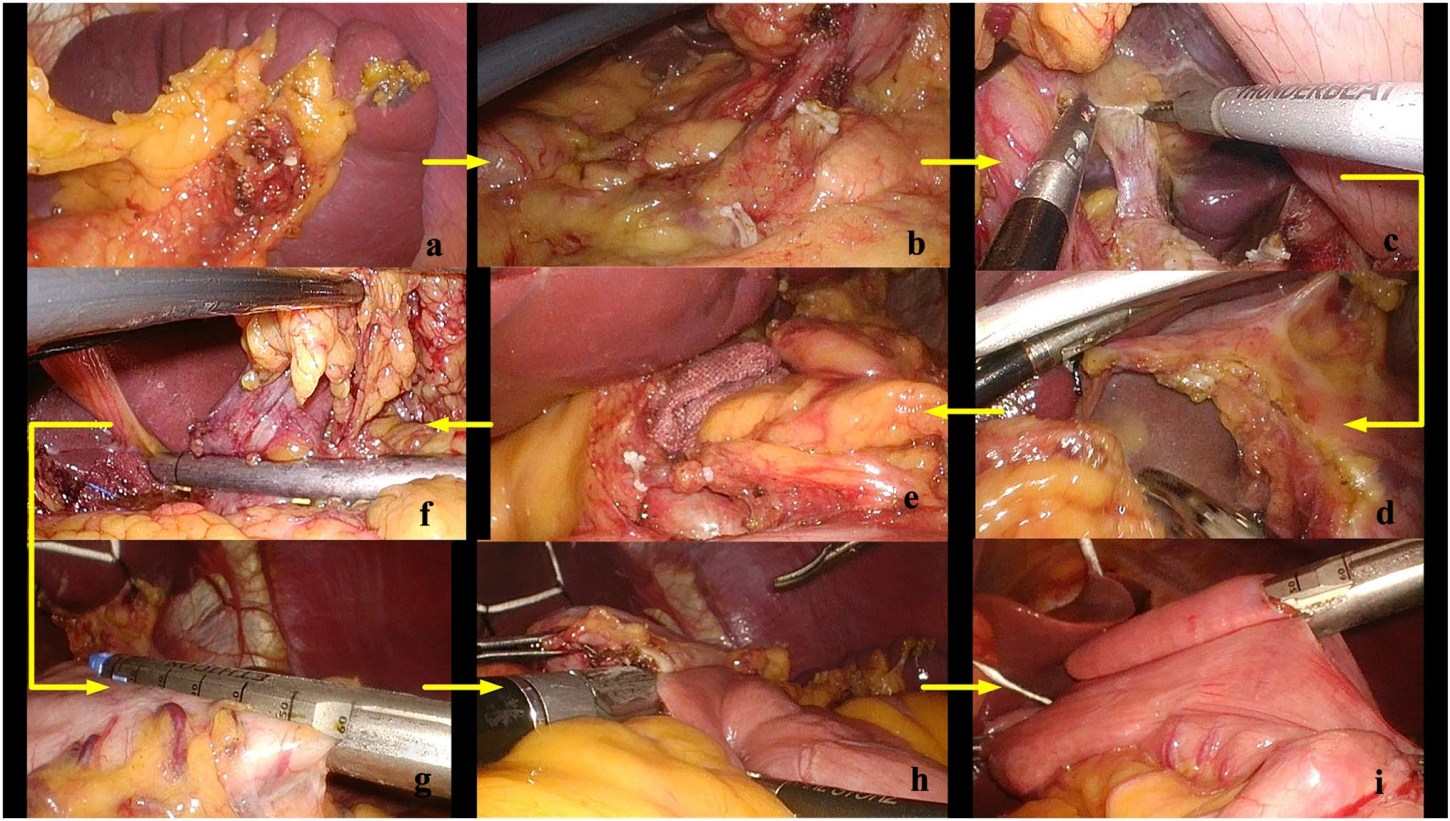
3D laparoscopic Surgical procedures. **a** The greater curvature of the stomach was dissociated, the left gastroepiploic artery and vein (LEGA&V) were dissected, and No.4sb and 4d were dissected; **b** The RGEA&V dissected, and No. 6 were dissected; **c** No. 7, 8a, 9, 11p, and 12a were dissected; **d** Dissociate the anterior wall of lesser curvature of stomach, and No.1 and 3 were dissected; **e** The RGA&V were dissected, and No. 5 were dissected; **f** The duodenum was dissected; **g** Determine the upper margin to separate the gastric wall; **h** Billroth II anastomosis; **i** Braun’s anastomosis

All patients who were enrolled underwent radical distal subtotal gastrectomy by using either the da Vinci Surgical Xi system (Intuitive Surgical) or 3D laparoscopy. D2 lymphadenectomy was performed for patients with advanced GC or any suspicion of nodal metastases. The specific surgical procedures are shown in Figs. 2 and 3.

### Statistical analysis

All data were analyzed by SPSS 24.0 statistical software. The mean ± SD (standard deviation) was used to represent the measurement data, and t test was used for comparison between groups. The chi-squared test (*χ*^2^ test) was used for comparison between groups of enumeration data, and Fisher exact probability method was used when applicable. Mann–Whitney *U* test was used for comparison between groups of grade data. When the baseline data of the two groups were inconsistent, the propensity score matching was used, and the 1:1 nearest neighbor matching method was applied. The matching tolerance was set to 0.02. All above test levels were considered statistically significant if *P* < 0.05.

## Result

### Intraoperative situation

The intraoperative bleeding in the TRDG group was less than that of the 3D-TLDG group (30.21 ± 13.78 ml vs. 41.44 ± 17.41 ml; *t* = − 4.322, *P* < 0.001); the preparation time (Stent placement and positioning time, docking time) of the TRDG group for intraoperative preparation was longer than that of the 3D-TLDG group (31.05 ± 4.93 min vs. 15.48 ± 2.43 min; *t* = 24.207, *P* < 0.001); the time required for the reconstruction of the digestive tract in the TRDG group was less than that of the 3D-TLDG group (32.67 ± 4.41 min vs. 39.78 ± 4.95 min; *t* = − 9.160, *P* < 0.001); the operation time of the TRDG group and the 3D-TLDG group were 234.01 ± 42.06 min and 227.62 ± 39.24 min, respectively, and there was no statistical difference (*t* = 0.950, *P* = 0.344). In addition, the number of LNs detected in the TRDG group (39.63 ± 15.77) was not different from that of the 3D-TLDG group (38.86 ± 11.43) (*t* = 0.337, *P* = 0.737). The rate of lymph node metastasis between the TRDG group and the 3D-TLDG group was 7.74% and 6.80%, respectively, and there was no statistical difference (*χ*^2^ = 1.875, *P* = 0.171) (Table 2).

**Table 2:** Comparison of intraoperative correlation between the two groups after PSM

	TRDG group (*n* = 73)	3D-TLDG group (*n* = 73)	*T*/*χ*^2^	*P*
Blood loss (ml)	30.21 ± 13.78	41.44 ± 17.41	− 4.322	< 0.001
Operation time (min)	234.01 ± 42.06	227.62 ± 39.24	0.950	0.344
Intraoperative preparation time (min)	31.05 ± 4.93	15.48 ± 2.43	24.207	< 0.001
Digestive tract reconstruction time (min)	32.67 ± 4.41	39.78 ± 4.95	− 9.160	< 0.001
Number of lymph nodes detected	39.63 ± 15.77	38.86 ± 11.43	0.337	0.737
Lymph node metastasis rate	7.74% (224/2893)	6.80% (193/2837)	1.875	0.171

### Postoperative situation

The TRDG group resumed ambulation earlier than the 3D-TLDG group (14.07 ± 8.97 h vs. 17.49 ± 5.98 h; *t* = − 2.714, *P* = 0.007); the TRDG group had less postoperative pain than the 3D-TLDG group (*Z* = − 2.735, *P* = 0.006); the postoperative anal exhaust time of the TRDG group was faster than the 3D-TLDG group (1.78 ± 0.79 d vs. 2.18 ± 0.79 d; *t* = − 3.050, *P* = 0.003); the TRDG group gave patients the time to remove the NGT earlier than the 3D-TLDG group (2.44 ± 0.91 days vs. 2.84 ± 1.28 days; *t* = − 2.159, *P* = 0.033); Days on liquid diet did not show a difference (4.47 ± 1.99 days vs. 4.67 ± 2.79 days; *t* = − 0.511, *P* = 0.610); the postoperative hospital stay in the TRDG group was shorter than the 3D-TLDG group (7.74 ± 3.15 days vs. 9.97 ± 3.23 days; *t* = − 4.231, *P* < 0.001); but the cost of hospitalization for patients in the TRDG group was higher than the 3D-TLDG group (RMB 125,615.82 ± 11,900.80 vs. 89,907.15 ± 17,147.19; *t* = 14.617, *P* < 0.001); The total incidence of postoperative complications in the two groups was 6.85% (5/73) and 9.59% (7/73), and there was no statistical difference (*χ*^2^ = 0.363, *P* = 0.547). All patients were cured and discharged after conservative treatment (Table 3).

**Table 3:** Comparison of postoperative correlation between the two groups after PSM

	TRDG group (*n* = 73)	3D-TLDG group (*n* = 73)	*T*/*Z*/*χ*^2^	*P*
Time to get out of bed after operation (h)	14.07 ± 8.97	17.49 ± 5.98	− 2.714	0.007
Postoperative exhaust time (days)	1.78 ± 0.79	2.18 ± 0.79	− 3.050	0.003
Postoperative NRS score				
0	6	4	− 2.735	0.006
1–3	57	44		
4–7	10	25		
8–10	0	0		
Postoperative gastric tube removal time (days)	2.44 ± 0.91	2.84 ± 1.28	− 2.159	0.033
Postoperative fluid feeding time (days)	4.47 ± 1.99	4.67 ± 2.79	− 0.511	0.610
Postoperative hospital stay (days)	7.74 ± 3.15	9.97 ± 3.23	− 4.231	< 0.001
Hospital costs (yuan)	125,615.82 ± 11,900.80	89,907.15 ± 17,147.19	14.617	< 0.001
Total postoperative complication rate	6.85% (5/73)	9.59% (7/73)	0.363	0.547

## Discussion

A number of studies have confirmed the efficacy and safety of laparoscopic radical gastrectomy [11–13]. With the innovation of technology, 3D laparoscopy provided the benefit of less intraoperative blood loss and a lesser occurrence of excessive bleeding than the conventional 2D laparoscopic gastrectomy [14]. At the same time, the da Vinci surgical system has also been increasingly recognized in gastrectomy [15]. In our study, the advantage of robotic gastrectomy in controlling intraoperative bleeding is consistent with Lee et al. [16]. The higher-definition naked-eye 3D visualization by the robotic surgery approach facilitates the identification of finer structures, and more flexible joint mobility, thus avoiding the corresponding injury. In addition, the robot has better intraoperative bleeding management measures than 3D laparoscopy, allowing the operator and the assistant to use more measures simultaneously to rapidly manage intraoperative bleeding, in addition to ultrasonic scalpels and vascular clips. Moreover, the mechanical arm is also more stable in its grip on the tissues and is less likely to cause damage to some of the tiny blood vessels caused by the retraction under the scope.

There is no significant difference in the overall operative time between the two groups in this study, and we have analyzed the different periods of the procedures separately. The robotic team spends more time in intraoperative preparation because the robotic surgery requires more time for the assistant to set up the robotic arm prior to operation, while the 3D laparoscopic equipment is easier to move and debug. Although the operation of robotic surgery does not require a high tacit understanding among the entire surgical team compared with laparoscopy, operators shall divert attention to control the exposure of the surgical field and adjust the viewing angle, particularly at the early stage of the robotic learning curve [17]. However, the time required for alimentary tract reconstruction in robotic surgery is significantly less than in 3D laparoscopy. The difference may be due to the robot's advantage in common opening sutures, which helps the operator to control the stitch spacing at various angles and improves the operators' confidence in endoscopic suturing. In our study, we found that the robotic suturing of the common opening could often be done easily and reliably without the assistance of an assistant to retract the bowel or stomach wall, especially in patients with smaller abdominal spaces. The above result is generally consistent with the finding of Ye et al. [6]. However, the advantages of robotic lymph node dissection reported by Shen et al. [18] were not found in this study. This may require more research on robotic gastrectomy.

The ERAS model applied after radical surgery for distal gastric cancer is mainly reflected in effective analgesia, encouraging early ambulation, early removal of NGT, thus reducing the occurrence of postoperative complications and allowing patients to recover earlier. In this study, both groups of patients received full ERAS, but a comparison showed that patients in the robotic surgery group had earlier postoperative ambulation, lower postoperative pain scores, shorter anal exhaust time, earlier NGT removal, and shorter hospital stay. The reduction of postoperative pain is attributed to the less traumatic nature of the robotic system and its more flexible instrumentation, which makes it easier for the surgeon to complete the liberation of tissue and leads less distraction to abdominal wall around the Trocar. Moreover, the robot can better maintain the anatomical level during the operation, make the separation of the perigastric mesenteric space more accurate, and reduce the damage to the perigastric mesentery, thereby reducing exudation and irritation [19]. Patients who have undergone robotic surgery have shorter postoperative hospital stay due to less discomfort after the operation [20]. As the anastomosis is the same in both groups, no significant difference has been observed in the time of resume diet postoperatively. However, due to the high cost of robotic equipment and maintenance, the hospitalization costs for patients in the robotic surgery group are significantly higher.

No significant difference in the incidence of postoperative complications between the two groups was found in this retrospective study, while the overall incidence of postoperative complications in this study is similar to or even lower than the results of studies by Yang et al. [21] and Ye et al. [22] without using the ERAS model, indicating that both robotic and 3D laparoscopy combined with ERAS for distal gastrectomy are safe and feasible, and will not increase the risk of postoperative complications for patients.

In conclusion, both TRDG and 3D-TLDG are safe and feasible under the ERAS protocol, and provide good short-term curative effects. Although robotic surgery costs more and requires longer preoperative preparation time, it is associated with less intraoperative bleeding and less time to reconstruct alimentary tract. The robotic surgery combined with ERAS also correlates with more expedited postoperative recover.

## Data Availability

QZ had full access to all the data in the study and takes responsibility for the integrity of the data and the accuracy of the data analysis.
